# Smartphone application use in commercial wild capture fisheries

**DOI:** 10.1007/s11160-022-09727-6

**Published:** 2022-09-15

**Authors:** Julia Calderwood

**Affiliations:** Marine Institute, Rinville, Oranmore, County Galway, H91 R673 Ireland

**Keywords:** Apps, Commercial fisheries, Mobile applications, Small scale fisheries, Smartphone

## Abstract

Smartphones are increasingly the most common type of mobile phone used throughout the world, offering users the ability to browse the internet and access mobile applications. Smartphones are also often equipped with high definition digital cameras, accelerometers, gyroscopes, and GPS. They can, therefore, facilitate the collection and dissemination of data, often through purpose designed applications (apps). As a result, numerous apps have been developed for use in wild capture fisheries. These apps have been designed for a number of purposes including for data collection, providing information to fishers, being linked to value chains and post-harvest practices and for uses linked to employment, legislation and safety. These apps are used across the world from large commercialized fisheries to small scale fisheries. In the latter, apps have the potential to bridge a technology gap, possibly replicating the functions of multiple pieces of hardware such as are used on larger vessels. This paper presents results from a narrative literature review to provide a synthesis of smart phone apps currently available for use by commercial fishers. 84 smartphone apps were identified as being currently available for use in commercial wild capture fisheries throughout the world. Smartphone apps were available for a number of uses but they were predominantly used for data collection and reporting. While this review provides an important overview of the extent of app use in commercial wild capture fisheries there remains potential for future work to improve understanding of how beneficial app use is and what it is that encourages app use and determines an app as being ‘successful’.

**Supplementary Information:**

The online version contains supplementary material available at 10.1007/s11160-022-09727-6.

## Introduction

The use of mobile phone technology has spread rapidly around the world in recent years and mobile devices are now a fixture of modern day life (Deloitte 2017; Taylor and Silver 2019). At the end of 2019 5.2 billion people (67% of the world’s population) were subscribed to mobile phone services with 3.8 billion of these users having access to mobile internet, and these figures are predicted to rise to 5.8 billion and 5 billion respectively by 2025 (GSM Association 2020). Smartphones are also increasingly the most common type of mobile phone used throughout the world. In 2018, for example, a median of 76% of adults in advanced economies owned a smartphone, compared to 17% having a non-smartphone, and 45% of adults in emerging economies owned a smartphone, compared to 33% having a non-smartphone (Taylor and Silver 2019).

Smartphones have additional features to basic mobile or cell phones and provide users with the ability to browse the internet and support mobile and web applications. Smartphones are also frequently equipped with high definition digital cameras, GPS, accelerometers and gyroscopes as standard meaning it is possible to collect multiple types of data with them (Galotto-Tebar et al. 2020; Gutowsky et al. 2013; Papenfuss et al. 2015). As a result smartphones are becoming increasingly important in facilitating the collection and dissemination of data, ultimately improving the transfer of and access to a wide variety of information (Abila et al. 2013; Petrik and Raemaekers 2018; Sahin and Yan 2013). Smartphone applications (apps) often facilitate such data collection and dissemination and the use and availability of apps is constantly increasing with 1.85 million apps being available for download in the iOS App Store and 2.56 million available through the Google Play store for Andriod users in 2020 (Iqbal 2021; Papenfuss et al. 2015). Thus with the general increased ownership of smartphones, the reducing costs of ICT (Information and Communication Technology) and increasing signal coverage and internet connectivity for smartphones, apps are being developed to provide solutions to address various environmental, social and economic problems (Montiel et al. 2020; Petrik and Raemaekers 2018; Zhao et al. 2016).

The potential for the use of smartphones in fisheries as a data acquisition tool, in addition to educational and outreach purposes, has been recognized for a number of years (Gutowsky et al. 2013). More general use of such technology in fisheries can be traced back to the early twenty-first century as smartphones became smaller and cheaper, while apps that were suitable for use in fisheries were concurrently developed during this period (FAO and WorldFish 2020). A proliferation of apps for use in sport and recreational fishing is evident with anglers taking advantage of smartphone technology and locational services to access information ranging from how to tie knots to the best baits for different target species, find nearby fishing locations and their weather and water conditions, keep track of personal catches and share these with other anglers (Bradley et al. 2019; Galotto-Tebar et al. 2020; Hudson 2016; Papenfuss et al. 2015; Sharma and Dhenuvakonda 2019; Venturelli et al. 2017; Walsh 2020). Indeed, reviews on the function and use of mobile phone technology in recreational fisheries have been previously conducted, often highlighting the potential use of such technology in aiding data collection from wide ranging and often remote locations (Cooke et al. 2021; Johnston et al. 2021; Papenfuss et al. 2015; Skov et al. 2021; Venturelli et al. 2017).

The use of apps in commercial fisheries has not been as extensively documented and does not appear to be as prevalent as in the recreational sector. For larger commercial operations this may be as a result of vessels already being well equipped with the technology and hardware required to plan and execute fishing operations, monitor marine traffic, communicate with other vessels and record catches. Despite this, the potential use of smartphone apps in commercial fisheries has been recognized. Increasingly, apps that address the following uses are being developed; (1) data collection devices, (2) providing access to marketing opportunities, (3) lifesaving tools, and (4) ways to communicate with the wider fleet (Marschke et al. 2020; Mohamed Shaffril et al. 2015). In terms of small scale fisheries (SSFs) smartphone apps could help bridge a technology gap, possibly replicating the functions of multiple pieces of hardware as found on the bridge of larger vessels. Further to this, due to the de-centralised nature and remoteness of many landing sites associated with SSFs they are often data deficient, especially in developing countries (Jeffers et al. 2019). In these instances smartphone applications represent a tool for collecting high volumes of on the ground data, which could help to improve understanding of the status of fish stocks as well as aid in evaluating fisheries management policies (Gorospe et al. 2016; Jeffers et al. 2019). Commercial uses of smartphone apps in fisheries are another possibility, having the potential to boost economic gains by providing real-time data on market prices and demand (Jensen 2007). Other than the economic gains that could be achieved through the use of smartphone applications there are also important community-orientated dimensions from information access and sharing that could be important and applicable in fisheries (Aricat and Ling 2018).

The multiple uses and benefits of smartphone applications in commercial wild capture fisheries, coupled with the increasing prevalence and use of smartphone technology, means there is much potential for multiple apps to be developed to add to those currently available to fishers. As such this paper provides a synthesis of smartphone apps currently available for use by commercial fishers. It reviews the purpose and use of highlighted smartphone apps, their functionality, the occasions in which they are used and adopted and the scale and extent of their use. This review provides a synthesis of information from a number of sources including scientific literature, grey literature and searches of app stores, to provide an overview of the current extent and purpose of smartphone app use in commercial wild capture fisheries. By improving understanding of the current state of smartphone app use in this sector this review can be built upon to consider how such applications could be developed, what is lacking in current apps and how this technology could be further utilized.

## Methods

A narrative literature review (Cronin et al. 2008; Ferrari 2015; Grant and Booth 2009) was performed to find examples of smartphone applications currently being used in commercial wild capture fisheries. In this context the main aim of the narrative literature review was to summarize information from both scientific literature and grey sources of information on current smartphone app use in commercial wild capture fisheries, identifying where geographically apps are used, in which fisheries and for what purpose. Academic databases were searched for reference to smartphone application use in fisheries (Supplementary Material 1). The reference lists of the returned literature were further searched as a method to supplement electronic searches and identify additional applications designed for use in commercial fishing operations that were otherwise missed. It was acknowledged, however, that reference to many of the smartphone apps that are used in fisheries is not made in peer-reviewed literature. Consequently, searches were also made in internet search engines in addition to app stores (Supplementary Material 1). Initially searches for the use of apps anywhere in the world were identified but searches were performed in English. It was recognized, however, that this may limit returns from searches and result in omissions of apps developed in non-English speaking countries. To capture a wider diversity of apps in use around the world searches were also made in Spanish and simplified Chinese (Supplementary Material 1), as along with English, Mandarin and Spanish, have the most native speakers in the world (Lane 2021).

Apps returned from the searches were examined to identify those relevant to commercial wild capture fisheries. Any apps returned by these searches that were not related to commercial fishing or not designed for use by commercial fishers were discounted (e.g. games, recreational fishing apps, species ID apps). Apps that might be used by fishers but were not specifically designed for their use were discounted, e.g. generic weather apps (i.e. Windy), navigation apps (i.e. Navionics), or communication apps (i.e. Whatsapp). Apps related to the fishing industry but not designed for use by fishers were also not included for further discussion, e.g. traceability apps that are used after fish are landed and processed. Any reference to apps currently in development but not widely available for use by fishers were also discounted.

Following both these sets of searches and filtering the apps were grouped based on their overall intended use, falling within five broad categories; Science, Knowledge and Data Gathering, Information Provision to Fishers, Value Chains and Post-Harvest, Employment Legislation and Safety, and App Suites. Where sufficient information was available a distinction was made between apps that were designed for use in a specific fishery or area versus those developed for more universal use. It was also identified whether there were any monetary costs associated with their use. Where available, additional information on the number of app users, the operating system of apps, the phone hardware utilized by apps and the development process of apps was also identified.

## Synthesis of apps available for commercial wild capture fishers

### Purpose of apps

**Fig. 1:**
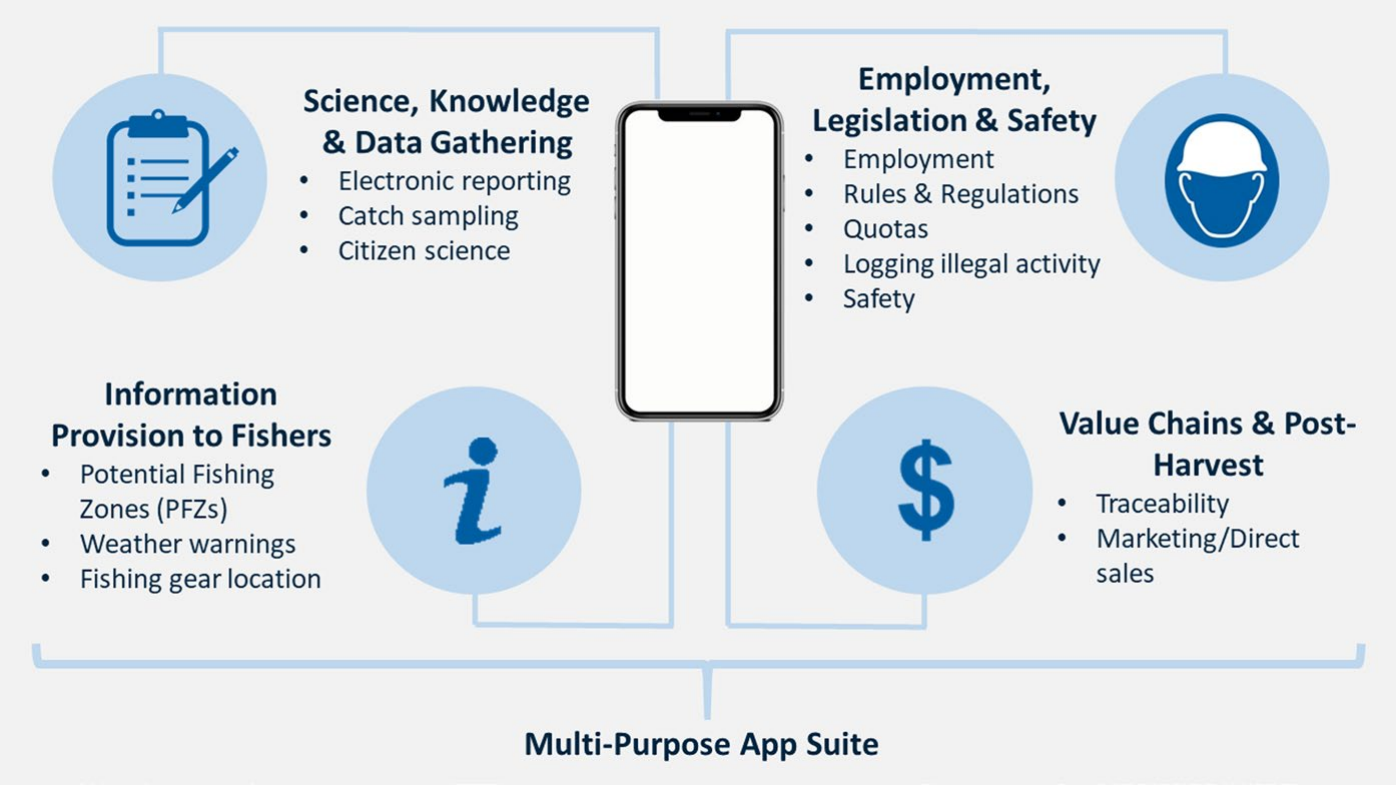
Diagram showing the five main categories of apps identified in the review along with examples of their use

A total of 84 apps were identified, 40 from searches of the literature with an additional 44 identified from mobile app stores. Apps were sorted under five main categories; (1) Science, Knowledge and Data gathering, (2) Information Provision to Fishers, (3) Value Chains and Post-Harvest, (4) Employment, Legislation and Safety and (5) Multi-Purpose App Suites, with a number of sub-categories identified under each (Fig. 1).

#### Science, knowledge and data gathering

The majority of apps identified (32 apps, 38%) fell under the category of ‘Science, Knowledge and Data Gathering’ with 30% (25) of all apps listed being categorized as ‘Electronic Reporting’ (Table 1, Supplementary Material 2). Electronic reporting apps included both those that facilitated mandatory reporting of catches to relevant authorities as well as methods of recording catches for personal records. Of these apps, eight were specific platforms to provide digital logbook data to relevant fisheries agencies. Examples include FisherMobile, a secure app developed to enable the reporting of commercial fishing activity by authorized fishers in Australia (NSW Government 2021), ELOG, an electronic log book for commercial fishers in Canada (Vericatch 2020a) and eCatch, another electronic logbook solution for New Zealand fishers (eCatch 2021). Another eight apps provide generic logbook solutions, offering electronic reporting solutions that were not designed for any specific fishery. These include eReporting, a customizable digital fleet recording app for use by governments, NGOs and communities to recording fishing data (Shellcatch 2019) and Deckhand Pro, which is a logbook app that utilizes a phone’s GPS to automatically map trips and activity in relation to recorded catch information (Fujita et al. 2018). The remaining apps in this sub-category provide ways to collect fisheries data that can be used by individual fishers or to support decision making within fisheries, including PescaData in Mexico and OurFish in Honduras, Belize and Myanmar (Frost 2017; PescaData 2020). Of these electronic reporting apps eight required payment for use, whether a one off payment to access the app or a subscription to regularly use the reporting software.

**Table 1:** A table showing the categories and sub categories of identified smartphone apps, example uses of these apps and number of each recorded. (Full details of each of the individual apps recorded can be found in Supplementary Material 2–6)

Category	Sub category	Example use	Total number
Science, knowledge and data gathering	Electronic Reporting	• Electronic logbooks • Personal record of daily catches and expenses • Catch recording linked to phone GPS to automatically record catch position	25
	Catch Sharing	• Facilitate voluntary catch avoidance schemes	2
	Citizen Science	• Record water quality data • Record litter at sea • Report tagged animals	3
	Additional Data Collection	• Bycatch data recording	2
	Total	**32**	
Information provision to fishers	Potential Fishing Zones (PFZ) / hotspots	• Disseminate PFZ advisories to fishers	3
	Fishing Gear Location	• Log and share location of ropeless gear	2
	Other	• Access information on seasonal closures	2
	Total	**7**	
Value chains and post-harvest	Traceability	• Enable end-to-end traceability in seafood supply chains	4
	Marketing/Direct Sales	• Establish direct links between fishers and consumers for seafood selling/buying	10
	Total	**14**	
Employment, legislation and safety	Employment	• Register crew details • Monitor crew health in relation to COVID-19	4
	Rules and Regulations	• Submission of required vessel reports • Provision of up to date regulations	7
	Quotas	• Check quota availability • Purchase or trade quotas	2
	Logging Illegal Activity	• Taking photos to report suspected illegal fishing activity	3
	Safety	• Submit reports on fishing accidents • Use phone GPS and accelerometer to determine vessel stability • Safety drill checklist	3
	Other	• Tracking applications for subsidy schemes	2
	Total	21	
App suites	–	• Suite of apps aimed at improving monitoring, traceability and transparency • Suite of apps to assist in reducing risks to natural hazards • Service platform linked to phone GPS to enable visualization of boat tracks, storing locations, sharing waypoints and accessing marine boundary information • Suite developed to aid digital traceability • Information provision from numerous sources including sea conditions, weather, rules and regulations etc	
	Total	**12**	

Other apps that fell under the category of Science, Knowledge and Data Gathering included apps that allowed vessels to share catch information with each other, with the aim of reducing catches of by-catch and quota restricted species (Table 1). These included BATmap, adopted by groundfish skippers in Scottish fisheries to avoid unwanted catches of cod and spurdog (Marshall et al. 2021), and eCatch which facilitates a 2-way sharing of logbook data between skippers in the USA (Merrifield et al. 2019). Three apps which facilitated citizen science among commercial fishers are also included, where fishers can contribute to scientific projects by collecting images of water surfaces to record turbidity and assist in monitoring data quality (Takemura et al. 2019), report tagged animals captured (Department of Primary Industries and Regional Development 2014) and record litter collected at sea (Lixo 2021).

#### Information provision to fishers

Seven apps were identified as providing information back to fishers, rather than collecting it from them, including three apps to help identify potential fishing zones (PFZs) and two helping to show the locations of static gear (Supplementary Material 3). All three apps detailing the location of potential fishing zones, as based on satellite data relating to sea surface temperature and water colour, were recorded as being used in Indian fisheries with the Machli app specifically providing information in eight Indian languages and the PFZ advisory app and mKrishi app being available in nine languages (Kiranmayi and Sharma 2020; Reliance Foundation, n.d.). Two apps were also identified as providing a platform for fishers to log and share the location of ropeless gear. While the Trap Tracker app is specifically designed to control EdgeTech’s ropeless fishing gear, Ropeless Fisher is a generic application that allows the position of ropeless gear to be logged throughout the world (EdgeTech 2021; Myers et al. 2019).

#### Value chains and post-harvest

In terms of post-harvest practices ten apps were designed to allow for direct sales from the fishing boat to the final consumer, all of which were developed for use in a specific fishery (Supplementary Material 4). Four of these apps were linked to traceability in fisheries, allowing fishers to capture and store information regarding catches to allow end-to-end traceability from harvest to consumption. Vericatch have developed Knowyour.fish, which can be customized to be used by any seafood business to help build transparent and sustainable supply chains, requiring payment for this service (Vericatch 2020b). The three other apps within this sub-category are developed for use in specific regions around the world including MPEDA Catch in India, TSER in Micronesia and eACDS in southeast Asia (Supplementary Material 4; KCS 2021; Siriraksophon et al. 2017; WCPFC 2020).

Apps relating to marketing and direct sales were more numerous than those linked to traceability, with ten apps falling under this sub-category (Table 1). Each of these ten apps were developed for use in specific regions and countries throughout the world to provide a route for direct sales between individual fishing boats and consumers. The aim of many of these apps is to provide a fair price for fishers. Fresh Fish Alert, an app for use in Sicily, Italy places an emphasis on sustainability with producers having to follow a strict set of social and environmental guidelines to list their catches on the app (Penca et al. 2021), while Blue Lobster in Denmark also emphasizes the low impact of its registered fishers (Blue Lobster 2020).

#### Employment, legislation and safety

The second largest category for apps was ‘Employment, Legislation and Safety’ (21 apps, 25%, Table 1, Supplementary Material 5) and included sub-categories of employment, rules and regulations, quotas, illegal activity and safety, with the majority of apps falling under these being developed for specific fisheries. Those linked to employment include facilitating the registration of crew, recording data required under Work Time Directives and even tracking and reporting test results of crew in relation to COVID-19 (Boyle 2020; Gorospe et al. 2016; Mendix 2021). The majority of these apps (7 out of the 21) were related to rules and regulations, providing information on local or national regulations and providing easy ways to provide necessary reporting in relation to these rules. Another two apps were specifically designed to keep fishers up to date with quota availability, FishRight in Sweden and NSS Mobile in Norway (Environmental Defense Fund 2019; Norges Sildesalgslag 2016). Three apps were also identified as helping fishers to log illegal fishing activity with DASE enabling app users to upload photos and locations of vessels fishing illegally to a central database where the evidence can be used by officials to catch and sanction perpetrators (Environmental Justice Foundation 2020). All three of the apps categorized as relating to vessels and crew safety have been designed for universal use. These include SCraMP, which utilizes the GPS and accelerometer on the smartphone to produce data related to a vessel’s stability, highlighting any threat of capsize for smaller vessels (Table S5; McCue 2013).

#### App suites

Finally, 12 app suites were identified where a single app provides multiple utilities for fishers all in one place (Table 1; Supplementary Material 6). The aim of these app suites varied from focusing on monitoring and management, including the ability to log catches, provide access to marketing opportunities, to providing fishers with navigational tools and information on weather and oceanographic conditions. Each of these suites was designed, at least originally, for use in a specific region. ABALOBI, an Android app originally developed in South African fisheries to improve monitoring, traceability and transparency in SSFs now has greater than 5000 downloads and its reach is growing.

### Operation and functionality

**Fig. 2:**
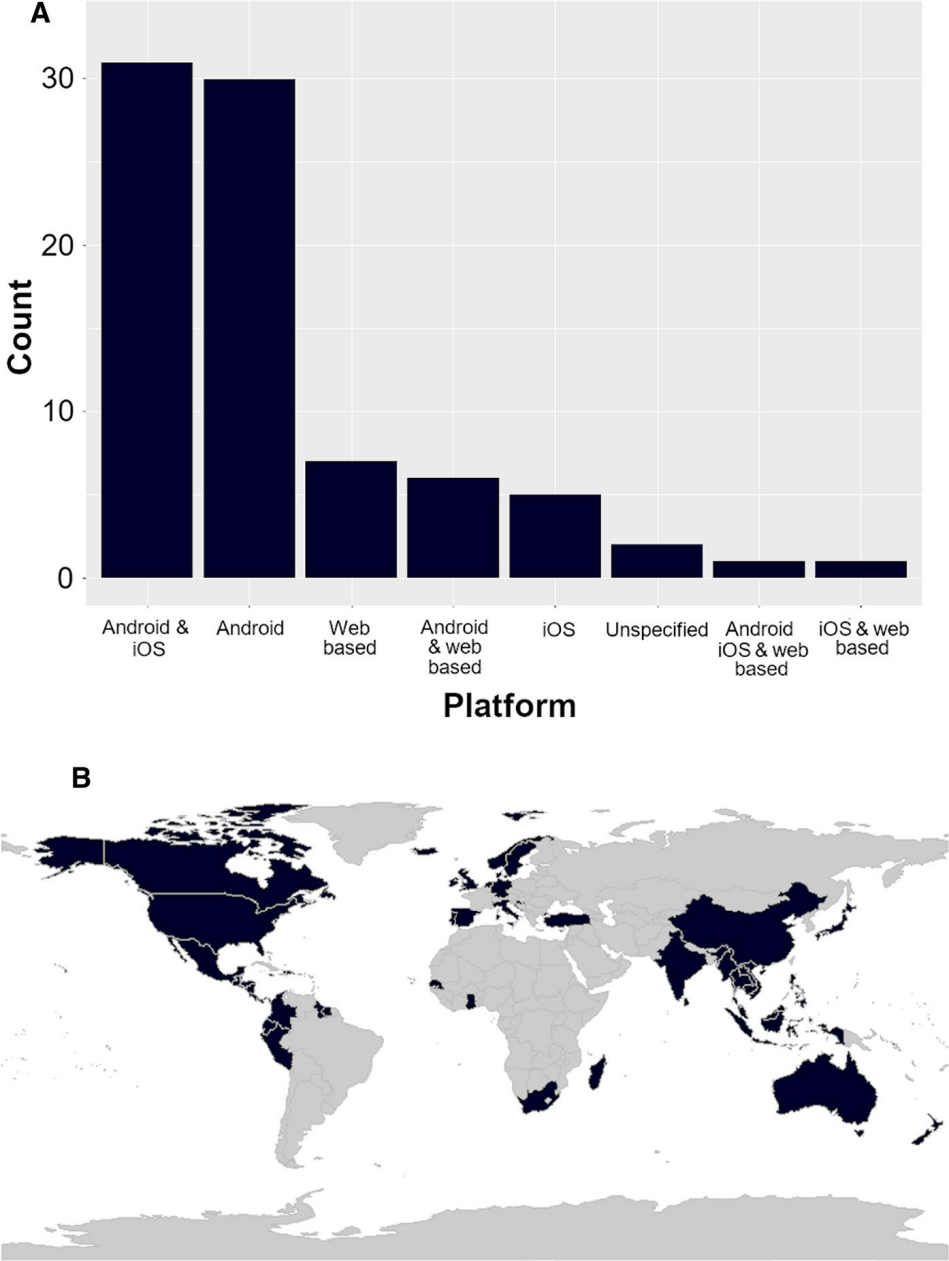
**A**. Barchart showing the platform upon which identified apps were available. **B**. A world map highlighting countries where identified apps were developed and intended for use (excluding apps that are available for worldwide use)

There are a number of recurring similarities across the apps in terms of their functionality, the range of operations they perform and the hardware they utilize on smartphones. The majority of apps identified are available across both Android and iOS platforms (31 apps), although 29 apps are solely available for Android compared to just five available only on iOS (Fig. 2a). An additional seven apps are accessed through the web rather than a specific platform, allowing for desktop use in addition to use on internet enabled smartphones.

A number of the apps utilize inbuilt hardware within smartphones to fulfil their objectives of use (Table 2). The inbuilt GPS in the phone was important for recording the location when data were recorded as well as for navigational purposes. Not all of the apps that required positional information relied on the phone GPS, with apps including ELOG and eCatch linking to vessel VMS or GPS systems to extract this information. The use of a phones camera was also important for many of the apps to not only upload pictures of catches (e.g. FishGIS), or to report vessels engaged in illegal activity (e.g. DASE) but to allow QR codes to be scanned to log certain activity such as registering fishing (e.g.WebControl Pesca).

**Table 2:** The hardware and functionality required by the apps presented in Supplementary Material 1–5

Category	Hardware and Functionality	Use	Example apps
Phone Hardware	Utilises phone GPS	Record location when entering/logging data	Deckhand Pro, FisherMobile, JOBEL, Afladagbókin, Bitácora Electrónica de Pesca, FishTagWA
		Use location for navigational purposes and visualising vessel tracks	FFMA, MFisheries, Odaku
	Utilises phone GPS, gyroscope and accelerometer	Records ships movements to determine if the vessel is unsteady and at risk of capsizing	SCraMP
	Utilise time/date information from phone	Automatic population of time/date information at time of recording data	FishTagWA, Bitácora Electrónica de Pesca
	Utilises phone camera	To take and upload pictures to provide supporting information as required by the app (e.g. photos of catches/by-caught species, photos of illegal fishing activity, evidence from accidents at sea, to contribute to citizen science projects)	FishGIS, FishTagWA, NetGuard, DASE, Mazu, Denuncias Pesquería del Caribe en Guatemala, FISHER, HydroColor, Mar sem Lixo
		Use camera to scan QR codes linked to app (e.g. securely and simply registering fishing activity and catches, logging marine litter drop off points)	WebControl Pesca, Mar sem Lixo
	Utilises phone microphone	Record voice note to describe fishing accidents at sea	FISHER
Usability	Simple icons	Quickly and simply access and input data using clear recognisable icons and pictures to select in the app	OurFish, FishGIS, ABALOBI, FEWER
	Drop-down menus	Reduce need for typed input	FISHER, mFish
	Multilingual	App provided in multiple languages to suit a range of end users	EFICE, M-Catch, Sagara, Caribbean Fishery, Oadaku
	Offline use	If the smartphone is out of cellular signal range data is saved on the app and uploaded later once within range	PescaData, JOBEL, eCatch, Afladagbókin
		Can be used offline to fill in data forms, only requires internet signal to send data	TSER
Additional Requirements	Links to vessel VMS or GPRS system	Instead of relying on a phones GPS an app may link into positional hardware already on-board a fishing vessel to provide locational information	ELOG, eCatch
	In-built payment options	App uses monetary payment software to enable selling of fish direct to the public	Blue Lobster, Straight Off The Boat, SaiEkvira Fishery
	Restricted access	Only approved users are able to access the app	FisherMobile, eCatch, Nelpin, NOAA Fish online
		Users must set up an account and access the app using their user ID and password	mFish, SkippersMate Pro, Straight Off The Boat
Development	Configurable/customizable software	Customizable application so that it can be tailored to meet the needs of multiple agencies	FisheriesApp, eReporting, OlracMDDL
	Open source	Open source code supplied so that others can use an app in a format that suits their fishery	ODK, Shiny4SelfReporting

A number of recurring themes related to the usability of apps are also highlighted in Table 2. One of the most important aspects of functionality for apps used by fishers appears to be ease and speed of use, to enable use while working at sea in often challenging environments. The use of simple icons and drop down menus were often recognized as being important to allow quick input, while reducing the need for typed input and the possibility for errors. The ability to still use apps when offline, with data being uploaded once back in range of signal, was important for many of the apps, and is particularly applicable to offshore use as is associated with fishing by larger vessels. Password protection was also evident in a number of the apps to restrict who was able to use them, often only allowing access to registered fishers or vessels within a particular fleet (e.g. FisherMobile, Nelphin and mFish). Finally, there were examples of apps that weren’t designed for use in any one specific fishery where the software was customizable so that it could be tailored to the needs of multiple end users operating in different fisheries. Some apps went a step further than this and have supplied open source code so that anyone is able to access this information and develop an app based on these examples, such as with ODK and Shiny4SelfReporting.

### Geographic spread of app use and extent of use

Apps were designed for use across the globe. Excluding apps that were designed for universal use across multiple fisheries, Fig. 2b shows the spread of app use from fisheries across the world. Of those apps designed to be used in specific countries nine were linked to use in India and nine in the USA, with six linked to Indonesia and three to the UK. All of the other countries highlighted in the map were linked to less than three apps.

A number of the apps detailed in Supplementary Material 2 to 6 have published data regarding the number of users and downloads of the apps. These numbers, where available ranged from > 10 to > 10,000 downloads, varying depending on the scale of intended use of the app, the apps usefulness, as well as depending on the fisheries where they are utilized. Laut Nusantara, an app suite designed for use in Indonesia had the largest number of recorded downloads at over 50,000 (Handayani 2021). Indonesia has a coastline extending to 54,716 km with a marine fleet comprising of 620,830 vessels in 2012 (FAO 2014). Thus, when considering the large fishing fleet this particular app suite is being used by approximately 8% of vessels. Nelpin, another app designed for use in Indonesia, providing fishers with information including estimated fishing area maps (Wiyono et al. 2018), hasn’t achieved such great user numbers in comparison to the size of the fishery with approximately 10,000 downloads. Machli, an app providing information on PFZs in Indian fisheries, reported 10,000 users, as did FFMA (Fisher Friend Mobile Application), another Indian app providing a decision support tool to fishers (Anabel et al. 2018; Reliance Foundation 2021). In India, however, there are approximately 1.9 million people employed in marine capture fisheries with approximately 264,000 vessels involved in this sector in 2014 so app use still remains relatively low (~ 4% of vessels) compared to the numbers involved in fishing (FAO 2006, 2019b).

Blue Lobster and FishLine, two apps used for direct sales between fishing vessels and consumers have over 5000 downloads (Blue Lobster 2020). Although it is not possible to determine how many of the app users are customers as opposed to fishers these numbers still give some indication of the success of these apps as many of the other apps categorized as marketing and direct sales had fewer than 100 downloads. Downloads of around 100 or less are not always an indication, however, that an app has limited uptake in a fishery. In 2008 there were 1529 fishing vessels registered with the Icelandic Maritime Administration (FAO 2010), so over 100 downloads of the Afladagbókin app used for electronic reporting of smaller vessels in Iceland could indicate a significant part of the fleet using the app (FISKISTOFA 2020). In Portugal, however, where there were 8046 fishing vessels registered in the fleet in 2015 (FAO 2017) around 10 downloads of the Mar sem Lixo app to report marine litter represents very low usage amongst the fleet (Lixo 2021).

Downloads of an app can give an indication of the potential extent of use but other apps have published their own user statistics that provide a better indication of use. For example, mFish, a catch reporting app for Indonesian fishers, has 14,000 users accessing the system each month (Fujita et al. 2018). While the Straight Off The Boat app currently has fishers deliver fresh catches to over 650 customers in the South West of England (Straight off the Boat 2021). More than four million reports have also been submitted through the VESL platform, which is an integrated hub that supports catch reporting to ten different agencies across fisheries in the USA (Bluefin Data 2018).

In terms of other reported successes FVDrills and SCrAMP, two apps designed for use in the USA to improve safety on board fishing vessels, now have users across the world (McCue 2013). Researchers evaluating MKrishi, which is another Indian app helping fishers to identify Potential Fishing Zones, have found the use of the app can save fishers up to 30% on diesel costs due to better targeting of catches (Singh 2018). The provision of weather and oceanographic information by the app is also reported to have contributed to no loss of life of fishers using the system following the Ockhi cyclone in 2017 (Singh 2018). The importance of the BATmap app, and its’ contributions to environmental sustainability, was also recognized, after being awarded The Sustainability Award in the 2021 Fishing News Awards for UK and Irish fishing industries (Fishing News 2021).

## Discussion

There are numerous apps available for use throughout commercial wild capture fishing operations, from SSFs to large industrialized fisheries, as identified from a review of the literature and search of the web and app stores. This, demonstrates there is an appetite for such technology in the fishing industry.

### Purpose and use of apps

In terms of the purpose of the apps, one of the main uses identified in this review was facilitating science, knowledge and data gathering, and specifically electronic reporting. This includes facilitating mandatory reporting via electronic logbooks, to support dockside catch sampling, or to provide more informal records for use by fishers and fisheries managers. Information gathering is recognized as an important part of fisheries management (FAO 1997; Jacquet et al. 2010) and fisheries monitoring is undoubtedly transitioning from paper records and occasional dockside sampling to more digital and traceable solutions (Boenish et al. 2020). Logbook data remains the primary source of landings information for many fisheries around the world (Russo et al. 2016). Electronic logbooks have replaced paper records in many of these fisheries in recent decades with numerous fisheries management authorities now utilizing electronic logbook software to streamline fisheries data collections (e.g. National ELOGS initiative in Canada (Government of Canada 2022), Electronic Recording and Reporting systems as required in EU fisheries within the European Fisheries Control Technologies Framework (European Commission 2009, 2011; Russo et al. 2016), compulsory use of e-logs in selected Commonwealth fisheries in Australia (Government 2015)). Despite established electronic reporting software being available in desktop applications that can be installed on-board fishing vessel computers, smartphone apps still appear to provide a suitable alternative platform for this purpose (Southern 2017). Where available, download statistics for apps providing electronic logbook solutions for specific fisheries, indicate reasonable uptake in relation to the number of vessels in each fleet. As such, investment in these resources by fisheries management bodies could be worthwhile where such applications are not currently available.

Catch reporting solutions could also be especially useful for smaller scale vessels that don’t necessarily have the hardware on-board to submit catch data via desktop applications. The Afladagbókin app in Iceland provides an example of providing alternative electronic logbook solutions for smaller vessels not subject to mandatory electronic reporting (FISKISTOFA 2020). Indeed, app technology shows promise for providing options for fishers to record catch data where electronic reporting isn’t mandatory. This can be for personal records to enable fishers to track catches alongside costs and sales prices, or to bolster official records and better inform management. Certainly having evidence of income and expenses in fisheries in Belize and Honduras has been transformative thanks to the use of the OurFish app, providing evidence of a financial history that can be used to access regulated financial supports and services (Irby 2017). As the importance of involving fishers in data collection is increasingly recognized (Haggan et al. 2007; Prescott et al. 2016), and as the need to find low cost solutions to achieve this are sought, there is great potential for the use of catch recording apps in fisheries at a variety of scales.

For authorities that do not want to invest in developing their own logbook and catch recording solutions a number of applications already exist that are customizable to different fleets, although there is often a cost associated with their use that has to be absorbed either by the management authority or the individual fisher. Open-source software options do exist to facilitate data collection in fisheries that may have limited financial resources to invest in bespoke data collection apps (de Graaf et al. 2017; Jeffers et al. 2019; Oviedo and Bursztyn 2017). These mobile technologies also show promise for more generic data collection, in remote areas and data deficient fisheries (Gorospe et al. 2016). Smartphone apps can be particularly useful to reduce the on-the-ground man power required for data collection, reduce the time between data collection and data use and reduce data management processes (Oviedo and Bursztyn 2017). Open source software provides an ideal starting point for more dispersed and small scale fisheries to explore how smartphone applications could be of benefit.

In addition to catch reporting, apps can make it easier to meet other reporting requirements such as registering crew or filing license applications. In these instances app technology can be especially beneficial in SSFs, which are often characterised by the use of seasonal and migrant workers, low quality infrastructure and remote working and landing sites, which make collection of such data otherwise difficult (Petrik and Raemaekers 2018). Data on downloads and use of such apps were limited so it was difficult to discern if they are used as much as those related to catch reporting. Where reporting on crew or vessel activity is mandatory, however, it may be useful to combine catch reporting with other required paperwork on one platform to make the process more streamlined and encourage all relevant submissions electronically. Apps can also be used to facilitate non mandatory data collection, for example, apps designed to be used by fishers to contribute to citizen science projects and apps for fishers to document and report illegal fishing activity. Where available, however, download figures for such apps are much lower than corresponding catch reporting apps. Even with illegal fishing being a widespread phenomenon with a broad range of socioeconomic and environmental impacts (Belhabib and Le Billon 2020), there may be little motivation for fishers to take the time to report such non mandatory data. While fishers can provide a valuable source of information they may not have the time or inclination to do so when out fishing, even when there are apps that facilitate such reporting. Issues surrounding trust, in relation to how collected data may be used and who it may be used by, could also influence uptake of apps (Calderwood et al. 2021a). When developing such apps it is, therefore, important to consider what may motivate a fisher to take the time to submit the relevant information to determine likely uptake.

After ‘Science, Knowledge and Data Gathering’ most apps fell into the category of ‘Employment, Legislation and Safety’. While some of these apps facilitate data collection in relation to filing required paperwork or logging illegal activity many of the apps under this category provide data to fishers regarding rules and regulations and quotas. Much of this type of information is provided on-line but fishers may not have access to devices other than smartphones to access this, especially once working at sea. Providing this data in one place via an app, rather than requiring searches of the internet, could be useful although where available, download statistics did not generally indicate high usage of such apps. This is in contrast to apps providing data on PFZs to fishers, that were associated with relatively high usage statistics. This may be because this is information that is not easily accessible elsewhere on the internet, with these apps compiling climatic and oceanographic data to provide information on the most likely location of catches. Thus for information that is easy to access on-line, or not directly linked to daily fishing operations a dedicated information provision app may not be necessary.

Innovation in distribution channels and direct sales apps can provide a way to boost incomes for fishers, and to fish to meet market demands, reducing waste and costs (Penca et al. 2021). Accelerated use of ICT solutions for use in distribution has indeed been seen in the SSF sector as a result of the COVID-19 pandemic with a need for many fishers to expand their customer bases due to restaurant closures (Penca et al. 2021). Whether the demand for such direct sales apps continues post pandemic remains to be seen but many of these examples show how the industry can adopt smartphone technology themselves. Having access to up to date market information can also be important to reduce price dispersion and waste, ultimately increasing fishers’ profits (Jensen 2007; Purcell et al. 2017). Although many of the apps that provide such information do so as part of a range of data provision, so it is difficult to determine if app downloads and usage is related to the provision of economic data alone. As more emphasis is put on the need for sustainable fisheries, apps can help to increase transparency along the supply chain and aid in reducing overexploitation of fish stocks, but also provide an incentive for use by fishers. Users of such apps could be rewarded by proving they are adopting less damaging and wasteful fishing practices and track this through the supply chain, ultimately increasing customer confidence in their purchases (Iles 2007; Purcell et al. 2017). There is certainly a lot more potential for the future development of apps in the field of traceability using blockchain technology for example (Ferreira Cruz and Rosado Da Cruz 2020). There has indeed been success trialing blockchain technology in tuna fisheries in Indonesia, utilizing smartphones so that traceability can start from the point of catch (Howson 2020; Provenance 2016). With sustainable fishing certification schemes requiring more evidence that fisheries are acting in ecologically responsible manners smartphone apps linked to traceability could provide some of this. As such the use of such apps may be of particular interest to producer organizations and fishery co-operatives in addition to fish processors and buyers.

This paper highlights the diversity of apps available for use by those working in wild-capture fisheries. Other apps may also be used by fishers that were not specifically designed for use by this industry and as such have not been included in this summary. But there are many more generic apps that could be useful to fishers including weather and tide apps for example. With so many options of available applications, app suites can also be useful, providing multiple services and sources of information in one place, rather than being designed for one specific utility. By providing multiple functions in one place app suites can also act to meet larger objectives than a single use app. ABALOBI, an African based social enterprise for example, describes their app suite as ‘A co-designed and fisher-driven mobile app suite to transform fisheries governance from hook to cook’ (Abalobi 2021). By collecting reliable data, including fishers experiential knowledge, ABALOBI aims to develop local resource stewardship and build socio-ecological resilience as well as facilitating mobilization by congregating small-scale fisher action towards value chain upgrading (Nthane et al. 2020). App developers may, therefore, want to consider multiple needs of a fishery when designing associated apps rather than producing a range of individual apps that may not all get used by any one fisher.

### Functionality

To encourage use of apps there are a number of considerations to make regarding their functionality. This includes the platform fishers are most likely to use to access mobile apps. The majority of apps featured are available on Android, which is likely to reflect the phones being used by fishers accessing these apps. While mobile phone and ICT use is increasing throughout the world there remains a digital divide and access to and the use of mobile phone tech is not equal across or between nations (FAO and WorldFish 2020). Consideration may need to be given to how old the smartphones being used to access the apps are and what versions of Android and iOS are being used. This was true of the Indonesian FishGIS app, as although smartphones are common in Indonesia many rely on older operating systems and it was therefore necessary to ensure the app would function on both old and new operating systems (Makino and Wells, 2020). Options do also exist to utilize more basic mobile phones in fisheries, with some fishers being able to access information through SMS queries, such as with EFMIS (Enhanced Fish Market Information Service) in Kenya (Abila et al. 2013). In recreational fisheries data collection via SMS has certainly provided foundations upon which smartphone apps have later been developed (Baker and Oeschger 2011; Gutowsky et al. 2013). In many instances these options may have been the precursors to the smartphone apps in use today in commercial wild capture fisheries and in some instances these kind of solutions may still benefit fishers, providing a bridge until more individuals have access to smartphone technology. For many of the apps reviewed, however, the functionality offered by a smartphone was essential for their use. This includes apps that utilize the cameras, GPS and accelerometers on smartphones.

Many of the apps utilized a phone’s GPS (e.g. to record location), accelerometer (e.g. to monitor vessel stability) or camera (e.g. to record illegal activity) to enable full app functionality. With increasing sophistication of smartphone technology the use of smartphone apps can provide alternatives to expensive equipment that fishers may not otherwise be able to afford (Aricat and Ling 2018). The lack of space or electricity on smaller vessels and within SSFs also mean smartphone apps have great potential. For many being able to check information while at sea may not be possible without a mobile phone. A number of the apps highlighted by this paper, especially the app suites for example, provide weather forecasts to help in reducing risks fishers take at sea. Smartphone apps might also replace other devices such as acoustic fish finders by providing advice on where fish are likely to be found, replacing plotters by utilizing a phone’s GPS and utilizing accelerometers to provide vessel stability information. Where space or cost may be prohibitive for using other hardware on board fishing vessel consideration should, therefore, be given to how smartphone technology can be used to provide fishers with necessary resources while operating at sea.

Another consideration regarding the functionality of apps is their overall ease of use. Fishers using the Nelpin app for example, were happy to use it because it provided them with the information they needed while having a good display and being easy to operate (Wiyono et al. 2018). The OurFish app was developed and tested with end users to ensure it was easy to navigate using icons and images. This also allowed the OurFish app to be easily adjusted to different languages and address varying literacy rates amongst its users (Irby 2017). The use of simple icons or pictures should, therefore, be considered to aid in the speed at which data can be recorded while also overcoming issues regarding digital skills, language and literacy levels. With regard to reducing input errors the use of drop down menus with set inputs can be beneficial. The Deckhand app requires forms to be fully completed, otherwise the app won’t shut down, providing an example of a method that can be used to ensure data entry is complete prior to submission (Fujita et al. 2018). Digital collection can still be prone to errors but those associated with time, date and locational data can be reduced by relying on smartphones to auto-populate required data fields. Phone GPS is used, for example, to record the location of recorded catches in the Deckhand Pro, eACDS, FishGIS and OlracMDDL apps (Fujita et al. 2018; Olrac 2021; Siriraksophon et al. 2017; Takemura et al. 2019).

While facilitating ease of use and data entry can be important considerations of apps, how any collected data is used and stored is another important consideration. A number of the apps identified had restricted use, with users having to register accounts or be approved by relevant management authorities before accessing apps. When commercially sensitive information is being collected app users may need to be reassured that data will be stored securely and not shared beyond approved users. It has been recognized that there is a culture of secrecy in many fisheries (Evans and Weninger 2014), this coupled with a lack of trust amongst fishers, in addition to concern over how data collection agencies may use data, could result in a lack of uptake of apps if there are no guarantees about who can access recorded data (Calderwood et al. 2021a; Ramirez-Sanchez and Pinkerton 2009).

A further key requirement identified across many of the apps was the ability to use the apps when there is limited network connectivity and for apps to be usable offline, with any data recorded being uploaded once within range of signal. Such functionality is an attractive solution that was prominent across many of the apps from both SSFs and larger commercial fisheries. It could be argued as being essential for fishers using apps when working offshore or in remote locations, although larger vessels may be able to access the internet through satellite or long-range WIFI solutions. This again highlights that the facilities available to fishers on board vessels in different fisheries needs to be considered when designing relevant smartphone applications.

Interestingly eight of the apps identified as associated with catch reporting carried a monetary cost. This was the largest proportion of any of the other app categories, indicating that this may be a service fishers are willing to pay for, possibly linked to the mandatory nature of this type of data collection. It is also possible that even with the added costs of needing a smartphone and associated data charges, data collection apps still have potential in reducing time and cost when compared to paper based methods (de Graaf et al. 2017). Paying for apps may, however, remain a barrier for some. This also raises the question of who is paying for the development and hosting of the majority of apps that are freely available. By 2016 Android had paid over $20 billion to app developers with Apple having paid $70 billion, and although a simple utility app can cost as little as $1000 to develop, this cost has to be absorbed somewhere (TADCO 2020). In terms of electronic reporting there may be benefits for those that require the data. Other apps have been developed by NGOs, researchers and fishers themselves, each of whom may be able to cover development and data storage costs to ensure the objectives of the apps are met. App development costs may be minimal in terms of expected benefits, such as streamlining processes or empowering fishers. Development costs were, however, cited as one of the reasons for the retirement of iCatch, an app developed to help vessels < 10 m in England and Wales to record catches and help submit shellfish returns (FD 2021). Despite industry support for the app, the time and costs to continually develop the app to meet industry requirements, in addition to cloud server costs, and a lack of adequate financial support proved detrimental for iCatch. This demonstrates that even for apps that provide innovative solutions to existing problems within a fishery and which are regularly used by fishers, there need to be considerations for continual funding if such apps are to be kept up to date and remain useable and active.

While many of the apps highlighted in this paper were free to use a number were also open source, with the source code that builds the application being freely available. This can enable others to adapt apps that already exist to their own fisheries rather than starting from scratch in the app development. This could certainly save efforts being duplicated to produce apps for use in different fisheries that essentially have the same purpose. Using open source code to build an app may not be all that simple, however, especially if there is limited documentation or support provided by the original developers (Heron et al. 2013). Making apps flexible so that they can be used in multiple fisheries and geographic locations could, however, remain beneficial, rather than multiple apps with the exact same features and functionality being developed simultaneously. As such, reviews like this are important to see what has already been developed and where gaps in app technology for use in fisheries still exist.

### Locations in which apps are adopted

This review shows use of apps throughout the world, with higher numbers developed for use in India, USA, Indonesia and the UK. With their extensive coastlines and significant fisheries the large numbers of individual working in fisheries in Indonesia and India could reflect the larger number of apps available in these countries (FAO 2014, 2019b). In terms of individuals and vessels involved, the fisheries in the USA and UK are smaller with approximately 27,000 commercial fishing vessels with licenses to operate in the United States EEZ (economic exclusion zone), there are just approximately 6300 registered fishing vessels in the UK fishing fleet (FAO 2016, 2019a). The prevalence of apps available in these two locations could be due to searching in English, or could indicate that the size of a fishery isn’t necessarily related to the design and use of apps.

Despite only searching the literature in three languages, apps were identified for use in countries across the world for use in both small scale and larger fisheries. This demonstrates there is a wide appetite for app use in a variety of fisheries. While the majority of the apps were designed for use in a specific country or fishery there were a number of apps that were designed for more generic use and could be adopted for use anywhere, in some instances with additional customization options that would make them more suitable for different fisheries. Other apps that were originally designed for use in one location also now have users elsewhere including the SCraMP and FvDrills safety app, originally designed for US fisheries, which now have users around the world (McCue 2013) and the ABALOBI app suite is trialing use of the application outside of South Africa where is originated (ABALOBI 2021). User language may be an issue, however, if apps are to be used in multiple locations. Already a number of the apps are available in multiple languages but this should be a consideration if developing new apps and may remain a limiting factor for apps to have greater transferability between fisheries and increased global appeal.

Despite the spread of apps being utilized across many different fisheries it remains difficult to determine the exact scale of use of all of the apps identified, as although downloads can be used as a metric for uptake it does not equate to a measure of regular use, and in many instances download statistics are not publically available. Download statistics alone do also not indicate how often an app is used once downloaded onto a smartphone. For app developers, determining uptake and usage rates may be important to determine the success of an app and to confirm its use. How to measure uptake and success may be something that needs to be considered during initial app development stages, especially if evidence of uptake is something funders of an app may require. Analyzing app users in relation to numbers in the fishery is also something to consider rather than just raw numbers as the size of fishing fleets varies considerable throughout the world. To be successful, however, an app does not have to be used every day by thousands of fishers. If a small group of fishers find an app to be useful in their everyday life this could be deemed as a success.

### Co-design examples

For a number of the apps the importance of a co-design process was noted. Co-design refers to designers, or in the case of apps software developers, working together with those not trained in software development to design and develop applications (Fox et al. 2022; Sanders and Stappers 2008). Co-design could refer to managers or scientists working together with app designers but industry stakeholders and fishers can also be included in this process. Involving end users can be particularly important to guarantee their needs are understood and met and that their experiences and expertise are considered to ensure an optimal app is produced (Steen et al. 2011). Input from stakeholders on app design can occur throughout its development, from initially identifying a problem that an app can address through to getting feedback during initial testing and roll out. The demand for an app like Fisher Friend, which provides a decision support tool for fishers in India specifically related to risks associated with poor weather, grew following a number of devastating tsunamis in India. Once a need for the app was identified a participatory approach, involving fishers as well as other key stakeholders from the Fisheries Department, Indian Meteorological Department, INCOIS (Indian National Centre for Ocean Information Services), MSSRF (M. S. Swaminathan Research Foundation) and Qualcomm, was used to develop the app (Anabel et al. 2018). From the inception of the Fisher Friend app over 1000 fishers were also involved in a pilot to test the platform and provide feedback on their experiences using it. Subsequently over a four year period the app went through over 40 versions migrating from a CDMA (code division multiple access) platform to an Android app, finally being available in 9 local languages (Anabel et al. 2018). Likewise, the OurFish app, designed to record catch data in Honduras, Myanmar and Belize, also went through a number of iterations with the first version of the app being scrapped due to it being an unintuitive tool not really meeting the requirements of its end users (Irby 2017). Field testing was also noted as being an important part of the process of developing the PescaData app, with feedback from fishers being built into the current product, which facilitates the recording of daily catches and expenses (Fulton 2021; PescaData 2020).

Development of the ABALOBI platform, an initiative born out of collaboration between a number of bodies and stakeholder groups, was an iterative and bottom up approach (Abalobi 2021; Nthane et al. 2020; Petrik and Raemaekers 2018). Approximately 100 fishers were involved in testing and development of the app, to ensure that the final product both empowered fishers within the market chain while fitting in with reporting and policy requirements (Petrik and Raemaekers 2018). Researchers from the University of Aberdeen drove the development of BATmap, a catch sharing platform for use by Scottish fishers to assist in avoiding unwanted catches, while working together with an Alaskan IT developer and the fishing industry (Marshall et al. 2021). A user-centred, co-design process was again used so that the software would be easy and intuitive to use, while reflecting fisher’s tolerance for sharing information and meet their needs for data security (Calderwood et al. 2021b). A pilot phase was then conducted with 13 vessels, operating in ICES area 6a (the west coast of Scotland), testing how well the app functioned in terms of assisting vessels to avoid unwanted catches (Marshall et al. 2021).

The usefulness of all of the apps is reliant in part on the development process and there is a need to balance the usability of the app with its intended purpose, such as to generate data (Teacher et al. 2013). Co-design approaches can be important to firstly identify fishers needs and gaps in the market for new apps. Participatory approaches including fishers and other relevant stakeholders in addition to app developers can then be important to ensure an app actually meets the requirements of all end users while being easy to understand and use. Pilot testing is another important stage in the app development process with a number of the apps being tested for a significant period of time, and undergoing adjustments, prior to final release. For those developing new apps for use in fisheries appropriate time and resources have to be given for these design and development stages to increase the likelihood that developed apps will meet end user requirements.

### Review limitations

While this paper gives an overview of the types of smartphone apps available for commercial wild capture there are limitations to this study. Firstly, as searches were performed in English, Spanish and Chinese, it is possible that apps for use in other languages were missed. While apps were found for use in countries that do not speak these three languages, it is likely that apps that operate in the search languages may be more predominantly represented in this review. The apps identified were also spread across countries all around the world but it is likely that there are omissions and it cannot be assumed that smartphone apps are only used in commercial fisheries in the countries identified in this review.

As a number of reviews of the use of app technology in recreational fisheries have been conducted this review has instead focused on their use in commercial fisheries, as comprehensive information on this topic has been lacking to date. In some instances, there may be cross over between recreational and commercial fisheries. This is especially true in the United States where landings from recreational fisheries can be substantial and thus there are requirements to manage recreational fisheries accordingly and include then in fisheries management plans (Jiorle et al. 2016). As such, apps may be developed to meet requirements of both fishing sectors. Any apps that could be used by both commercial and recreational fishers, such as Fish Rules which provides information on the rules and regulations regarding all saltwater fishing in the USA, are included in the review. Apps that were designed solely for use in recreational fisheries were, however, excluded from this review. It remains possible, however, that apps that could be utilized in both commercial and recreational fisheries were missed by not specifically searching for recreational fisheries in the literature.

One of the other challenges with this review is that the sources of information available for each of the available apps vary quite considerably. Sources of information on smartphone applications for use in commercial wild capture fisheries were found in peer reviewed literature but also grey literature including magazine and news articles, dedicated app websites, app developer websites and app stores. With few peer reviewed papers published regarding the use of smartphone apps in commercial fisheries the use of grey literature makes an important and invaluable contributions to this review (Paez 2017). For some apps there were multiple sources of information with specific details regarding their use and development, but for others the information available was more limited. The varying quantity and quality of information for each smartphone app identified makes it difficult to provide a direct comparison of each of the apps and their full functionality and range of use. The review does, however, provide an important overview of smartphone app use in commercial wild capture fisheries, providing a significant contribution to the literature in this field, demonstrating how apps are currently being used in this field.

### Avenues for future work

As detailed in the previous section there are a number of limitations to this study, which highlight a need for a further systematic review on the topic. This would allow for more informative comparisons between apps to be made to determine those that are used most and which could be deemed more successful in terms of meeting needs of fishers and being popular to use. It would be of benefit to extend this work to better determine the uptake rates of smartphone apps in fisheries, digging deeper to find out how often apps are used and by how many individuals or vessels, rather than just exploring download statistics as a metric of use or proliferation in a fishery. This avenue of work could also determine the rate of app uptake to determine trends and ascertain if there is growing demand for these applications. More work could also be done to determine how successful apps have been in meeting the needs of fishers and providing useful products. This would likely require not only a review of the literature but time spent interviewing app developers and users. While the development process of apps has been touched on in this review a more detailed review on smartphone app development in commercial fisheries could also be of benefit to highlight lessons learnt and detail best-practise for future app development.

## Conclusions

This research shows that smartphone applications have a place in commercial wild-capture fisheries to meet a number of uses from data collection to improving safety at sea for fishers, as well as being used in supply chains and marketing. Apps directly related to fishing activity generally had greater uptake compared to apps offering other functionality, such as non-mandatory reporting. While larger commercial vessels may have the space and infrastructure for numerous pieces of hardware that can be used to inform their fishing practices, this is not always the case for smaller vessels, and smartphone apps can potentially replicate the functionality of some of this hardware. There is still a place for smartphone apps for use by fishers operating on these larger vessels and apps have been specifically designed for use in these fisheries, including catch sharing and reporting applications. In many instances co-design has been recognized as being important to ensure apps meet the demands of the final users. Ease of use and low data requirements are key considerations to encourage uptake of mobile applications in fisheries. While numerous apps are currently available across fisheries around the world there is potential for continued development in this field. Already, apps that were originally developed for one specific fishery are being extended to more universal applications. As seen with the apps that were developed in in response to challenges that arose from COVID-19, apps can also be quickly developed to meet suddenly arising issues in fisheries, demonstrating further potential. While acknowledging that there does remain a digital divide and smartphone apps do not provide universal solutions to all fisheries, there is currently wide ranging use of apps throughout fisheries around the world, and as smartphone technology advances and becomes cheaper such apps are likely to be continued to be used and developed. Before developing new apps, however, it is worth exploring if apps available in other fisheries or areas could be applied elsewhere. Where new apps are developed consideration should be given to the needs of end users in terms of app purpose and functionality. Ways of monitoring app usage and user experience would also be beneficial to aid future app development.

## Supplementary Information

Below is the link to the electronic supplementary material.

Supplementary file1 (DOCX 64 KB)

## Data Availability

The datasets of mobile apps generated during the current study are available from the corresponding author upon request.
