# Supplementary material for: Smartphone application use in commercial wild capture fisheries
Source: Rev Fish Biol Fish. 2022 Sep 15;32(4):1063–83. doi: 10.1007/s11160-022-09727-6 (PMC9476459; doi:10.1007/s11160-022-09727-6)
Supplement: Supplementary file 1 — Supplementary file1 (DOCX 64 KB) [file 11160_2022_9727_MOESM1_ESM.docx]

Supplementary Material 1: Table detailing the databases, search engines and app stores and the search terms used within them in English, Spanish and Simplified Chinese to search smartphone applications used in wild capture fisheries

| **Search Location** | **Search Terms: English** | **Search Terms: Spanish** | **Search Terms: Simplified Chinese** |
| --- | --- | --- | --- |
| Web of Science, Scopus, Google Scholar, Google | app* OR mobile phone app* OR smartphone app* AND fish OR fishery OR fisheries OR fishing OR commercial fishing OR fishing industry OR small scale fishery OR smart fishing –angler -angling | aplicación* OR aplicación para teléfono móvil* OR aplicación para teléfono inteligente Y pescado OR pesca OR pesca OR pesca OR pesca comercial OR industria pesquera OR pesca a pequeña escala OR pesca inteligente –pescador-pesca | 应用程序  OR 手机应用  OR 智能手机应用  AND 鱼  OR 渔业 OR钓鱼 OR 商业捕 OR渔业 OR 小规模渔业 OR智能钓鱼 -垂钓者 -钓鱼 |
| Google Play Store and Apple App Store | Fish, Fishery, Fisheries, Fishing, Commercial Fishing, Fishing industry, Small scale fishery, Smart fishing | Pez, Pesqueria, Pesquerias, Pesca, Pesca commercial, Industria pesquera, Pesca a pequeña escala, Pesca inteligente | 鱼, 渔业, 渔业, 钓鱼, 商业捕鱼, 渔业, 小规模捕鱼, 智能钓鱼 |

Supplementary Material 2: Table showing apps categorized as facilitating science, knowledge & data gathering († denotes apps developed for generic use across locations and fisheries, $ denotes apps that are not available to use free of charge, user numbers identified alongside app platform where this information was available)

| **SCIENCE, KNOWLEDGE & DATA GATHERING** | | | | |
| --- | --- | --- | --- | --- |
| **Electronic Reporting** | | | | |
| **App Name** | **Country of use** | **Purpose** | **Platform** | **References** |
| ODK (open data kit) †($) | Worldwide | A free and open-source software designed for quick, accurate, offline data collection. Has been utilized for data collection in a number of different fisheries. | Android and web app | (de Graaf et al., 2017; Jeffers et al., 2019; ODK, 2021; Oviedo & Bursztyn, 2017) |
| Hapi fis, Hapi Pipol | Solomon Islands | A platform for local enumerators to collect information on fish caught and sold at market and submit to a centralized database to facilitate near real time management decisions. | Android | (Pomeroy & Yang, 2015; WWF, 2014) |
| OurFish | Honduras, Belize, Myanmar | App for fishers to track catches with numbers and sizes of fish easily recorded using simple icons | Android (>1000) | (Campbell et al., 2021; Frost, 2017; Irby, 2017) |
| Deckhand Pro †$ | Worldwide (developed in Australia) | Electronic logbook app, utilizing GPS to automatically map trips and activity in the recording device to supplement inputted information regarding catch, effort, bycatch etc. | iOS | (Fujita et al., 2018) |
| FishGIS | Indonesia | Fishers take photos of their catch and upload with additional information such as location, gear used, species etc. Additional information can be uploaded regarding garbage and illegal fishing activity. | Android and iOS | (Takemura et al., 2019) |
| PescaData | Mexico | App to record daily catches and expenses. | Android and iOS (>500) | (PescaData, 2020) |
| ORFISH logbooks † | EU | App that allows fishers and/or scientific observers to input data into logbook software. Inputted data can be reviewed by fishers in near real time. | Android and web app | (Orfish, n.d.) |
| WebControl Pesca | Mexico | Used as part of the Community Administration Monitoring Program allowing fishers to register via use of QR codes before tracking when they fish, how much they catch and where they sell it and at what price. | Android app | (Environmental Defense Fund, 2019) |
| FisherMobile | Australia | A secure app developed to enable the reporting of commercial fishing activity by authorized fishers | Web based app | (NSW Government, 2021) |
| ELOG | Canada | Electronic log book that allows commercial fishing catch and effort information to be entered and transmitted electronically | Web based app | (Vericatch, 2020a) |
| FisheriesApp †$ | Worldwide | Configurable software that enables the collection and transmission of fisheries data. | Android (>500) | (Vericatch, 2020b) |
| eReporting †$ | Worldwide, trialed in Puerto Rico | A customizable digital fleet reporting app for use by governments, NGOs and communities to record fishing data. | Android and iOS (>1000) | (Shellcatch, 2019) |
| CyFIS | Cyprus | An application developed for the recording and reporting of catches and fishing activities from both professional and amateur fishers. | Android and iOS (>500) |  |
| Mofi | Germany | An app designed for commercial vessels to automatically log position as sea and record fishing operations. | Android and iOS (>500) |  |
| eCatch $ | New Zealand | An electronic logbook for recording and submitting catch data in commercial fisheries. | Android and iOS (>500) | (eCatch, 2021) |
| FISHLog †$ | Worldwide | Electronic catch reporting software which allows of the integrations of national and regional fisheries. | Android and web based app | (CLS Applications, n.d.) |
| mFish | Indonesia | Allows fishers to enter catch data and then gain access on weather, fish prices and fishing best practice | Android and web based app | (Fujita et al., 2018) |
| OlracMDDL (Mobile Dynamic Data Logger) †$ | Worldwide | A user friendly system which allows fishers to report fishing activities whilst at sea, monitoring and recording catch and effort data while recording gear properties and fishing methods, environmental data and crew information. The app can use a smartphones built-in GPS to record positional information or link to externally connected GOS devices. | Compatible with all operating systems | (Olrac, 2021) |
| Afladagbókin | Iceland | An app to replace paper records for smaller vessels that are exempt from mandatory electronic reporting. The app allows catch and bycatch information to be submitted to the Directorate of Fisheries. Catch information can be recorded while at sea and uploaded later if signal is not available at time of recording. | Android and iOS (>500) | (FISKISTOFA, 2020) |
| Bitácora Electrónica de Pesca | Ecuador | An electronic fishing log app which automatically detects the date and location of fishing activity to increase accuracy of reported catch data. Data is submitted to the ministry of aquaculture and fisheries. | Android (>100) | (Ministerio de Producción, Comercio Exterior, n.d.) |
| BAGS (Elektronik Seyir Defteri Mobil Uygulaması) | Turkey | Electronic Logbook Mobile Application linked to Fishing Vessel Monitoring Systems for reporting to the General Directorate of Fishing and Fishery Products | Android (>500) | (General Directorate of Fisheries and Aquaculture, n.d.) |
| EFICE M-Catch | Netherlands | A web based online logbook that can be accessed via mobile devices | Web based | (Efice, n.d.) |
| Shiny4SelfReport | Worldwide | A customisable open-source application to provide a tool for self-reporting data in small-scale fisheries | Web based | (Eurico Mesquita Noleto-Filho et al., 2021) |
| JOBEL $ | Canada | Electronic logbook to allow data to be submitted to Fisheries and Oceans Canada (Pêches et Océans Canada) | Android, iOS and Web-based | (JOBEL, 2016) |
| eTrips Mobile | USA | Standard Atlantic Fisheries Information System (SAFIS) providing a platform for Atlantic coast harvesters, dealers and anglers. | Android, iOS and web-based | (Program, 2020)s |
| **Catch Sharing** | | | | |
| **App Name** | **Country of use** | **Purpose** | **Platform** | **References** |
| BATmap | Scotland | An app to facilitate a voluntary catch avoidance scheme, whereby participating skippers record hauls of unwanted species in ‘rea-time’ with alerts being sent to other participating vessels if agreed thresholds of catches are exceeded in any area. | Android and iOS (>10) | (Marshall et al., 2021) |
| eCatch | USA | eCatch allows users to capture, visualize and share logbook data with other vessels in a 2-way sharing relationship. | iOS | (Merrifield et al., 2019) |
| **Citizen Science** | | | | |
| **App Name** | **Country of use** | **Purpose** | **Platform** | **References** |
| HydroColor | Japan and Indonesia | Smartphone used by fishers to collect images of water surface to record turbidity to assist in monitoring water quality | Android and iOS (>1000) | (Takemura et al., 2019) |
| FishtagWA | Australia | App that allows reporting of tagged lobsters, crabs and finfish to the Department of Fisheries to help with research and stock assessments. | Android and iOS | (Department of Primary Industries and Regional Development, 2014) |
| Mar sem Lixo | Portugal | Vessels, which are members of the 'Fishing for a Sea Without Rubbish' project in mainland Portugal can use the app to record the location of marine litter collection, documenting this by uploading photos of the litter onto the platform. The app can also be used to record when the collected litter is deposited at collection sites back on land. | Android and iOS (>10) | (Lixo, 2021) |
| **Additional Data Collection** | | | | |
| **App Name** | **Country of use** | **Purpose** | **Platform** | **References** |
| Clean Catch | UK | App designed to gather data on accidental wildlife bycatch | Android and iOS | (*Clean Catch UK Launches New Wildlife Bycatch Reporting App*, n.d.) |
| NetGuard | Worldwide | App for both volunteers and fishermen to report catch and bycatch information as well as report NetGuard pingers underwater usage to prevent marine mammal entanglement | Android and iOS (>500) |  |

Supplementary Material 3: Table showing apps categorized as facilitating information provision to fishers († denotes apps developed for generic use across locations and fisheries, $ denotes apps that are not available to use free of charge, user numbers identified alongside app platform where this information was available)

| **INFORMATION PROVISION TO FISHERS** | | | | |
| --- | --- | --- | --- | --- |
| **Potential Fishing Zones (PFZ) / hotspots** | | | | |
| **App Name** | **Country of use** | **Purpose** | **Platform** | **References** |
| mKRISHI Fisheries | India | App detailing potential fishing zone (PFZ) locations based on satellite data on sea surface temperatures and water colour. Also provides weather forecasts, including wave height, wind speed and wind direction. | Android (>5000) | (Anon, 2017; Singh, 2018) |
| PFZ advisory | India | App used to disseminate PFZ advisories to fishers living in coastal areas. It also provides information on daily chlorophyll, sea temperature and turbidity measurements to assist in locating areas of abundant fish. | Android (>500) | (Kiranmayi & Sharma, 2020) |
| Machli | India | An app providing PFZ information to users in eight Indian languages. | Android (>10,000) | (Reliance Foundation, n.d.) |
| **Fishing Gear Location** | | | | |
| **App Name** | **Country of use** | **Purpose** | **Platform** | **References** |
| Ropeless Fisher † | World Wide | An app to log the location of ropeless gear (i.e. pots) and then displays this location to other fishermen operating within a ‘visibility radius’ to prevent gear entanglement. | Android (iOS in development) | (Desert Star Systems, n.d.; Myers et al., 2019) |
| Trap Tracker $ | USA | App based control of EdgeTech’s ropeless fishing system which also enables fishers to mark, map and record fishing trap information and share with others. | Android and iOS (>10) | (EdgeTech, 2021) |
| **Other** |  |  |  |  |
| **App Name** | **Country of use** | **Purpose** | **Platform** | **References** |
| Clima Pesca | Central America | An app produced by the Clima Pesca initiative, with the objective of providing information on the impacts of climate change on the sector, including current meteorological and oceanographic conditions. The project and app also aims to develop efforts that help to increase production and guarantee food security in the face of climate change. | Android and iOS (>100) |  |
| FiskInfo | Norway | An app that allows fishers registered with the coast guard to access information on area closures, location of certain fishing grounds, vessel locations etc. as well as navigational warnings, maps on ice concentrations, subsea installations and planned seismic activity. | Android and iOS (>1000) | (SINTEF, 2016) |

Supplementary Material 4: Table showing apps for use in marketing, direct sales and traceability († denotes apps developed for generic use across locations and fisheries, $ denotes apps that are not available to use free of charge, user numbers identified alongside app platform where this information was available)

| **VALUE CHAINS & POST-HARVEST** | | | | | | |
| --- | --- | --- | --- | --- | --- | --- |
| **Traceability** | | | | | | |
| **App Name** | | **Country of use** | | **Purpose** | **Platform** | **References** |
| Knowyour.fish †$ | | Worldwide | | An app to allow end-to-end traceability from harvester to consumer, utilizing verified catch data from the original harvester. | Android | (Vericatch, 2020c) |
| MPEDA Catch | | India | | An app to improve traceability and catch information in fisheries value chains. A mobile application allows fishers to log details about catches while a web based application allows exporters, data entry operators, field officers and administrators to add additional information as required. | Android, iOS and web (>100) | (KCS, n.d.) |
| TSER | | Micronesia | | A high seas transshipment electronic reporting system | Android, iOS and web (>10) | (WCPFC, 2020) |
| eACDS | | Southeast Asia | | An app to support an electronic catch documentation scheme. Fishers can record and report catches and vessel location in online or offline modes and later transmit information to the cloud server. Buyers and registered fish agents can also access the system to ensure all steps of the supply chain are recorded. | Android and iOS (>50) | (Siriraksophon et al., 2017) |
| **Marketing/Direct Sales** | | | | | | |
| **App Name** | **Country of use** | | **Purpose** | | **Platform** | **References** |
| Fresh Fish Alert | Italy | | Establishing a direct link between small scale fishers and consumers to enable virtual marketing of products caught within a strict set of social and environmental guidelines in Sicily | | Android and iOS | (Penca et al., 2021) |
| Blue Lobster | Denmark | | An app that allows chefs and private customers to order directly from low impact fishermen, receiving delivery of fish straight from the boat with fishers receiving a fair price | | Android and iOS (>5000) | (Blue Lobster, 2020) |
| SaiEkvira Fishery | India | | App to enable delivery of fresh fish and seafood from the boat to individual customers. | | Android and iOS (>1000) |  |
| Straight off the boat | UK | | An app to provide notifications when a local inshore fishing boat has fresh fish to sell. Also provides recipes and information on the species available to buy. | | Android and iOS (>100) | (*Straight off the Boat*, 2021) |
| FishLine | USA | | Fishers can post availability of seafood for free on the app allowing customers to find fresh local seafood to buy. Also provides a directory of fishers who will take orders for seafood. | | Android and iOS (>5000) |  |
| CatchDrive | Netherlands | | An app that offers customers the opportunity to purchase fish directly from fishing vessels while they are still at sea. | | Android (>50) |  |
| BahariFresh | Indonesia | | A platform for fishers as well as fish farmers and fishery entrepreneurs to sell fish and seafood direct to the consumer | | Android (>100) | (BahariFresh, 2021) |
| Pescamia | Italy | | An app that allows fishers to sell directly to consumers | | Android (>50) |  |
| Fishpedia | Sri Lanka | | A package of two apps, one for sellers and one for buyers, for selling fish directly from the fishing industry | | Android (>10) | (Fishpedia, n.d.) |
| PrizeFish | Adriatic | | An app that allows direct sales from fishers with an emphasis on locating fishers and sales closest to the buyer | | Android and iOS (>10) |  |

Supplementary Material 5: Table showing apps designed to meet employment, legislative and safety requirements († denotes apps developed for generic use across locations and fisheries, $ denotes apps that are not available to use free of charge, user numbers identified alongside app platform where this information was available)

| **EMPLOYMENT, LEGISLATION & SAFETY** | | | | |
| --- | --- | --- | --- | --- |
| **Employment** | | | | |
| **App Name** | **Country of use** | **Purpose** | **Platform** | **References** |
| Skippers Mate Pro | Ireland | An app developed to help industry meet the requirements of the Work Time Directive | Android and web app | (Boyle, 2020) |
| Sagara | India | An app to monitor the movements of fishing vessels and to register crew working on vessels as required by the Department of Fisheries | Android (>5000) | (Government of Kerala, n.d.) |
| FishR | Philippines | Fishers can register themselves and their fishing activities in a centralized database to save having to report to a central office. Access to additional government services such as health insurance provided as an incentive to register. | Android | (Gorospe et al., 2016) |
| KelvinCARE $ | USA (Alaska) | Application to track and report test results, monitor worker health and trigger fast responses if workers show COVID symptoms to prevent outbreaks of disease on vessels. | Web based app | (Mendix, n.d.) |
| **Rules and regulations** | | | | |
| **App Name** | **Country of use** | **Purpose** | **Platform** | **References** |
| Caribbean Fishery | Puerto Rico and U.S. Virgin Islands | The app provides information in English and Spanish on state regulations and federal regulations for the Exclusive Economic Zone (EEZ). | Android and iOS |  |
| DWG OP | New Zealand | An app to view the Deepwater Group’s operating procedures, 10 Commandments, Catch Limits and Quota information. | Andriod and iOS (>100) | (Deepwater Group, 2015) |
| SA Commercial Fishing Report | Australia | Free application to make mandatory reporting required for all South Australian commercial fishing license holders simple and easy | Android (>100) |  |
| NOAA Fish Online | USA | An app for operators of federally regulated fishing vessels who are required to submit VTRs (vessel trip reports) based on their fishing permits. | iOS and web app | (NOAA, n.d.) |
| VESL | USA | A hub integrated with 10 separate reporting agencies to allow necessary reporting to government with ease. | Android and iOS (>100) | (Bluefin Data, 2018) |
| Dualog SSN | Norway | An app for the mandatory reporting of arrival and departure information to Norwegian authorities and ports from fishing vessels via SafeSeaNet Norway. | Android (>100) |  |
| Fish Rules | USA | Simplifies saltwater fishing regulations into an easy to understand format based on location and season | Android and iOS (>500) | (Fish Rules, n.d.) |
| **Quotas** | | | | |
| **App Name** | **Country of use** | **Purpose** | **Platform** | **References** |
| FishRight | Sweden | Purpose built platform that allows fishers to check quota availability and purchase or trade quota with other vessels | Web based app | (Environmental Defense Fund, 2019) |
| NSS Mobile | Norway | An app to keep registered users updated on catch data, reported catches, auctions and quotas in the pelagic fishery. | Android and iOS (>100) | (Norges Sildesalgslag, 2016) |
| **Logging Illegal Activity** | | | | |
| **App Name** | **Country of use** | **Purpose** | **Platform** | **References** |

| DASE | Ghana | Allows canoe based fishers to upload photos and locations of vessels fishing illegally to a central database where the evidence can be used by government officials to catch and sanction perpetrators. | Smartphone app | (Environmental Justice Foundation, 2020; McVeigh, 2020) |
| --- | --- | --- | --- | --- |
| Mazu † | Worldwide | The app allows anyone to report vessels that might be fishing illegally or have histories of illegal fishing. | Android (>100) | (Benioff Ocean Initiative, n.d.) |
| Denuncias Pesquería del Caribe en Guatemala | Guatemala | The app allows reports on illegal actions that violate fisheries and aquaculture laws to the Directorate of Fisheries and Aquaculture Regulations | Android (>10) |  |
| **Safety** | | | | |
| **App Name** | **Country of use** | **Purpose** | **Platform** | **References** |
| FISHER (The Fishing Industry Safety Health Event Reporting) † | Worldwide | An app that acts as an input channel for the FISHER project, to submit reports on fishing accidents with the ability to submit GPS coordiantes and photos to back up other details. | Android and iOS (>50) | (FISH Safety Foundation Initiative, 2020) |
| SCraMP (Small craft monitoring program) † | Worldwide | Using the GPS and accelerometer technology within smart phones the app provide real time data related to vessel stability and threat of capsize for small vessels. | iOS | (McCue, 2013) |
| FvDrills † | Worldwide | A drill checklist app to enable crews on small vessels to conduct regular safety drills on-board vessels. | iOS | (McCue, 2013) |

| **Other** | | | | |
| --- | --- | --- | --- | --- |
| **App Name** | **Country of use** | **Purpose** | **Platform** | **References** |
| BKBD | India | An application used for tracking the status of applications for applying for subsidy schemes from the fisheries department for the government of Bihar. | Android (>100) |  |
| Xesnar | Spain | An app to enable centralized management and organization in shellfish fisheries, including coordinating daily quotas and working hours | Cloud (web) and android (>2000) | (Xesmar, 2019) |

Supplementary Material 6: Table showing app Suites ($ denotes apps that are not available to use free of charge, user numbers identified alongside app platform where this information was available)

| **APP SUITES** | | | | |
| --- | --- | --- | --- | --- |
| **App Name** | **Country of use** | **Purpose** | **Platform** | **References** |
| ABALOBI | Africa (and beyond) | A suite of apps aimed at improving monitoring, traceability and transparency in SSFs while facilitating the inclusion of fishers in government decision making as well as enabling them to develop their businesses. Apps include; ‘FISHER’ (personal log book with sharing options and safety at sea integrations), ‘MONITOR’ (digitized community catch monitoring at landing sites), ‘MANAGER’ (real-time fishery data and communications for co-management), ‘CO-OP’ (co-operative member and fleet management), ‘MARKET-PLACE’ (Empowerment in the value chain). | Android (>5000) | (Abalobi, n.d.; Petrik & Raemaekers, 2018) |
| FACTS (Fishing Activity and Catch Tracking System) $ | USA (Michigan and Maryland) | A set of data collection and management software modules including: ‘Hails’ (to send trip notifications), ‘Logbooks’ (send and record logbook data automatically), ‘Landings’ (used by dealers to monitor landings), ‘Quota’ (integrates with other modules to perform quota calculations), ‘Licensing’ (pay and renew licenses) | Web based app | (Electric Edge Systems Group, 2021) |
| WISE (wireless solutions for fisheries in Senegal) | Senegal | An app to provide fishers and fish processors with real-time market data (to enable negotiation of best prices for their fish), weather and ocean forecasts, SOS feature, warnings if fishers cross international boundaries of travel into restricted zones, provision of best practice information, access to affordable loans and mobile money. | Android (>50) | (Qualcomm wireless reach, 2018) |
| FFMA (Fisher Friend Mobile Application) | India | A decision support tool offering advice and information in a single platform including: GPS for navigation with risk zones marked, emergency contact, oceanographic information including wave height, currents, SST, weather information including wind speed and direction, information on government schemes to support fishers. | Android | (Anabel et al., 2018; Kiranmayi & Sharma, 2020) |
| Nelpin | Indonesia | Supports fishers through provision of a number of sources of information including an Estimated Fishing Area map, weather, tides, fish price information and fuel forecasts. | Android (>10,000) | (Wiyono et al., 2018) |
| FEWER (Fisheries Early Warning and Emergency Response) | Caribbean | An app to help small scale fishers reduce risks to hazards by providing; hazard warnings including storms, hurricanes and difficult sea conditions, weather checks, send and receive alerts, record and report damages after disasters and file missing person reports | Android (>100) | (*FEWER: The App Making Fisherfolk More Secure*, 2020) |
| MFisheries | Caribbean | A suite of resources including; navigation resources, weather forecasts, virtual marketplace and safety at sea resources | Android (>10) | (Caribbean ICT Research Programme, 2014) |
| Odaku | India | A service platform that links to GIS devices and includes features such visualizing tracks, storing location points and waypoints, sharing waypoints with groups of vessels, provide information on local borders. In addition to the GIS capabilities the app also, weather alerts in addition to providing a market place to buy and sell used boats. Available in eight local languages. | Android (>5000) | (Kiranmayi & Sharma, 2020) |
| MiPez | Columbia | An app to aid fishing communities in the Magdalena basin Columbia, to keep an inventory and control expenses, consumption, sales and profits as well as registering catches and fishing locations. Data feeds into the SIMA system which is used to document fishing activity in the area and learn about it's importance to the Colombian economy. | Android and iOS (>100) | (Conservancy, 2020) |
| TrazApp | Peru | A suite developed to aid digital traceability in artisanal fisheries. The platform obtains real time information on fishing activity, catches and sales with separate platforms available for skippers, vessel owners, operators of landings and merchants. | Android and iOS (>500) | (TrazApp, n.d.) |
| 渔业助手 (Fishery Assistant) | China | An app suite which provides a number of functions for fishers all in one place including positional information, sea conditions and weather at numerous fishing grounds, information linked to fisheries governance and regulations, a trading platform and a messaging platform | iOS |  |
| Laut Nusantara | Indonesia | Provision of multiple data sources including location of fish, weather data and fuel count in addition to features to make emergency calls. | Android (>50,000) | (Handayani, 2021) |

**References**

Abalobi. (n.d.). *ABALOBI*. Retrieved June 28, 2021, from http://abalobi.info/

Anabel, N. J., Velvizhi, S., & Suvitha, D. (2018). Fisher Friend Mobile Application: a decision support system for small scale fishers in India. *CSI Transactions on ICT*, *6*(3–4), 257–267. https://doi.org/10.1007/s40012-018-0212-1

Anon. (2017). mKRISHI Fisheries, a new mobile app to help fishermen. *Tech2*. https://www.firstpost.com/tech/news-analysis/mkrishi-fisheries-a-new-app-to-help-fishermen-3696171.html

BahariFresh. (2021). *BahariFresh*. https://baharifresh.com/

Benioff Ocean Initiative. (n.d.). *Mazu: a mobile application for information exchange on illegal fishing vessels*. Retrieved June 29, 2021, from https://oceanconference.un.org/commitments/?id=19469

Blue Lobster. (2020). *The first digital place to buy and sell low impact and fair seafood*. https://bluelobster.app/

Bluefin Data. (2018). *The smart, easy way to report: VESL simplifies the reporting process*. https://www.bluefindata.com/

Boyle, B. (2020). New App to Simplify Rule Change. *The Skipper*. https://theskipper.ie/new-app-to-simplify-rule-change/

Campbell, S. J., Jakub, R., Valdivia, A., Setiawan, H., Setiawan, A., Cox, C., Kiyo, A., Darman, Djafar, L. F., Rosa, E. de la, Suherfian, W., Yuliani, A., Kushardanto, H., Muawanah, U., Rukma, A., Alimi, T., & Box, S. (2021). Immediate impact of COVID-19 across tropical small-scale fishing communities. *Ocean and Coastal Management*, *200*, 105485. https://doi.org/10.1016/j.ocecoaman.2020.105485

Caribbean ICT Research Programme. (2014). *The Suite Applications*. https://www.cirp.org.tt/mfisheries/index.php/get-mfisheries

*Clean Catch UK launches new wildlife bycatch reporting app*. (n.d.). Retrieved June 28, 2021, from https://www.cleancatchuk.com/clean-catch-uk-launches-new-wildlife-bycatch-reporting-app/

CLS Applications. (n.d.). *FISHLOG: Electronic Catch Reporting Software*. https://fisheries.groupcls.com/wp-content/uploads/2020/07/FishLog-Datasheet-2020-final.pdf

Conservancy, T. N. (2020). *MiPez application protects biodiversity of the Magdalena River*. https://www.nature.org/es-us/sobre-tnc/donde-trabajamos/tnc-en-latinoamerica/colombia/mipez-aplicacion-pescadoras-agua-dulce/

de Graaf, G., Stamatopoulos, C., & Jarrett, T. (2017). *OPEN ARTFISH AND THE FAO ODK MOBILE PHONE APPLICATION: A TOOLKIT FOR SMALL-SCALE FISHERIES ROUTINE DATA COLLECTION*. http://www.fao.org/fishery/static/OpenArtfish/Toolkit.pdf

Deepwater Group. (2015). *Operational Procedures*. https://deepwatergroup.org/newsresources/resources/

Department of Primary Industries and Regional Development. (2014). *New Department of Fisheries fish tagging app launched*. http://www.fish.wa.gov.au/Pages/media_archive/New-Department-of-Fisheries-fish-tagging-app-launched.aspx

Desert Star Systems. (n.d.). *Ropeless Fisher App: Virtual Gear Marking and Smarter Practices for Ropeless Fishing*. Retrieved June 28, 2021, from https://www.desertstar.com/page/rope-less-fisher-app

eCatch. (2021). *eCatch: Electronic catch reporting made simple*. https://ecatch.co.nz/

EdgeTech. (2021). *Trap Tracker*. https://www.edgetech.com/product/trap-tracker/

Efice. (n.d.). *EFICE M-Catch*. Retrieved November 17, 2021, from https://efice.com/en/oplossingen-en/solutions-to-be-use-on-shore/efice-m-catch/

Electric Edge Systems Group. (2021). *FACTS: An Integrated Fishery Management Platform*. https://www.fisheryfacts.com/

Environmental Defense Fund. (2019). *Smart Boats and Networked Fisheries: New pathways to sustainable fishing in the digital age*. https://www.edf.org/sites/default/files/documents/SmartBoatVision.March2019.web_.pdf

Environmental Justice Foundation. (2020). *New phone app is effective weapon in Ghana’s fight against illegal fishing*. https://ejfoundation.org/news-media/new-phone-app-is-effective-weapon-in-ghanas-fight-against-illegal-fishing

Eurico Mesquita Noleto-Filho, Angelini, R., Steenbeek, J., & Carvalho, A. R. (2021). New, flexible and open-source fisheries self-reporting app: The Shiny4SelfReport. *SoftwareX*, *16*, 100843. https://doi.org/https://doi.org/10.1016/j.softx.2021.100843

*FEWER: The app making fisherfolk more secure*. (2020). The Caribbean Regional Track of the Pilot Programme for Climate Resilience. https://caribppcr.org.jm/fewer-the-app-making-fisherfolk-more-secure/

Fish Rules. (n.d.). *An innovative way to understand complex fishing regulations*. https://fishrulesapp.com/

FISH Safety Foundation Initiative. (2020). *FISHER App*. https://fisherproject.org/fisher-app

Fishpedia. (n.d.). *Fishpedia*. Retrieved September 23, 2021, from https://thefishpedia.com/#/landing

FISKISTOFA. (2020). *Afladagbókin - Smáforrit fyrir rafræna skráningu afla*. https://www.fiskistofa.is/umfiskistofu/frettir/afladagbokin-smaforrit-fyrir-rafraena-skraningu-afla

Frost, E. (2017). *There’s an App for That: Using Phones to Help Make Fishing Sustainable*. Smithsonian Ocean. https://ocean.si.edu/ocean-life/fish/theres-app-using-phones-help-make-fishing-sustainable

Fujita, R., Cusack, C., Karasik, R., Litsinger, E., Wirandi, W., Cunningham, E., Osman, L., & Amoros, S. (2018). *Technologies for Improving Fisheries Monitoring*. https://www.edf.org/sites/default/files/oceans/Technologies_for_Improving_Fisheries_Monitoring.pdf

General Directorate of Fisheries and Aquaculture. (n.d.). *The mobile application of the Electronic Log Book used by our fishermen has been published.* Retrieved September 23, 2021, from https://www.tarimorman.gov.tr/BSGM/Sayfalar/Detay.aspx?OgeId=87&Liste=Duyuru

Gorospe, K. D., Michaels, W., Pomeroy, R., Elvidge, C., Lynch, P., Wongbusarakum, S., & Brainard, R. E. (2016). The mobilization of science and technology fisheries innovations towards an ecosystem approach to fisheries management in the Coral Triangle and Southeast Asia. *Marine Policy*, *74*, 143–152. https://doi.org/10.1016/j.marpol.2016.09.014

Government of Kerala. (n.d.). *Portal for reporting and monitoring of fishing crafts and crews*. Retrieved May 28, 2021, from https://sagara.kerala.gov.in/MIS/public.php

Handayani, M. (2021). To make fishing easier, Minister Trengono developed the “Laut Nusantara” app. *VOI*. https://voi.id/zh/economy/41138/read

Irby, S. H. (2017). *Tracing Fish and Finances: Turning a catch data app into a life-changing tool for fishers.* Rare. https://rare.org/story/tracing-fish-and-finances/

Jeffers, V. F., Humber, F., Nohasiarivelo, T., Botosoamananto, R., & Anderson, L. G. (2019). Trialling the use of smartphones as a tool to address gaps in small-scale fisheries catch data in southwest Madagascar. *Marine Policy*, *99*(October 2018), 267–274. https://doi.org/10.1016/j.marpol.2018.10.040

JOBEL. (2016). *JOBEL: An innovative solution that replaces paper logbooks*. https://jobel.ca/

KCS. (n.d.). *Improving traceability and catch information in fisheries value chain for MPEDA with 15% rise in the exports*. Retrieved September 21, 2021, from https://www.kcsitglobal.com/storage/uploads/case_study/pdf/improving-traceability-and-catch-information-in-fisheries-value-chain-for-mpeda-with-15-rise-in-the-exports.pdf

Kiranmayi, D., & Sharma, A. (2020). Mobile apps and internet of things (IoT): A promising future for Indian fisheries and aquaculture sector. *Journal of Entomology and Zoology Studies*, *8*(1), 1659–1669. https://www.researchgate.net/publication/339844052

Lixo, A. P. por um M. S. (2021). *A Pesca por um Mar Sem Lixo*. http://www.marsemlixo.com/

Marshall, C. T., Macdonal, P., Torerson, E., Asare, J. L., & Turner, R. (2021). *Design, development and deployment of a software platform for real-time reporting in the west of Scotland demersal fleet. A study commisioned by Fisheries Innovataion Scotland (FIS)*. https://fiscot.org/wp-content/uploads/2021/05/FIS032.pdf

McCue, L. (2013). ON THE USE OF MOBILE APPS FOR IMPROVED MARITIME SAFETY. *International Conference IDS2013*, *22*, 1–15.

McVeigh, K. (2020). Ghanaians devastated by illegal fishing try hand at citizen sleuthing. *The Guardian*. https://www.theguardian.com/environment/2020/nov/11/fishing-app-launched-to-tackle-trawling-in-ghana

Mendix. (n.d.). *KelvinCARE Application Monitors Health of Alaska’s Offshore Workforce, Protects Vital Fishing Industry*. Retrieved June 28, 2021, from https://www.mendix.com/press/kelvincare-application-monitors-health-of-alaskas-offshore-workforce-protects-vital-fishing-industry/

Merrifield, M., Gleason, M., Bellquist, L., Kauer, K., Oberhoff, D., Burt, C., Reinecke, S., & Bell, M. (2019). eCatch: Enabling collaborative fisheries management with technology. *Ecological Informatics*, *52*, 82–93. https://doi.org/10.1016/j.ecoinf.2019.05.010

Ministerio de Producción, Comercio Exterior, I. y P. (n.d.). *BITÁCORA ELECTRÓNICA DE PESCA*. http://bitacora.produccion.gob.ec/bitacora/

Myers, H. J., Moore, M. J., Baumgartner, M. F., Brillant, S. W., Katona, S. K., Knowlton, A. R., Morissette, L., Pettis, H. M., Shester, G., & Werner, T. B. (2019). Ropeless fishing to prevent large whale entanglements: Ropeless Consortium report. *Marine Policy*, *107*. https://doi.org/10.1016/j.marpol.2019.103587

NOAA. (n.d.). *Electronic Vessel Trip Reporting Software Options*. Retrieved June 29, 2021, from https://www.fisheries.noaa.gov/new-england-mid-atlantic/resources-fishing/electronic-vessel-trip-reporting-software-options

Norges Sildesalgslag. (2016). *Application*. https://www.sildelaget.no/en/media/application/

NSW Government. (2021). *FishOnline - FisherMobile*. https://www.dpi.nsw.gov.au/fishing/commercial/services-for-fishers/fishonline/fishonline-commercial/fishermobile

ODK. (2021). *ODK Documentation*. 698. https://docs.getodk.org/_downloads/ODK-Documentation.pdf

Olrac. (2021). *Olrac Electronic Logbook Solutions*. https://elog.olsps.com/

Orfish. (n.d.). *Logbook application*. Retrieved June 28, 2021, from https://orfish.eu/help-logbook

Oviedo, A. F. P., & Bursztyn, M. (2017). Community-based monitoring of small-scale fisheries with digital devices in Brazilian Amazon. *Fisheries Management and Ecology*, *24*(4). https://doi.org/10.1111/fme.12231

Penca, J., Said, A., Cavallé, M., Pita, C., & Libralato, S. (2021). Sustainable small-scale fisheries markets in the Mediterranean: weaknesses and opportunities. *Maritime Studies*, 1–15. https://doi.org/10.1007/s40152-021-00222-5

PescaData. (2020). *PescaData*. https://pescadata.org/

Petrik, M., & Raemaekers, S. (2018). The case for supporting small-scale fisheries governance through ICT. *South African Institute of International Affairs*, *177*. https://www.africaportal.org/publications/case-supporting-small-scale-fisheries-governance-through-ict/

Pomeroy, R., & Yang, D. (2015). Selling and marketing fish in the Solomon Islands Business assets. *SPC Fisheries Newsletter*, *145*, 23–28. https://pacific-data.sprep.org/system/files/FishNews145_23_Pomeroy_0.pdf

Program, A. coastal cooperative statistics. (2020). *eTrips2 Mobiles: a SAFIS application*. https://www.accsp.org/wp-content/uploads/2020/06/eTripsM-brochure-_v2.1_FINAL.pdf

Qualcomm wireless reach. (2018). *Wireless Solutions for Fisheries in Senegal ( WISE )*. https://www.qualcomm.com/media/documents/files/senegal-wireless-access-for-fishery-wise.pdf

Reliance Foundation. (n.d.). *Machli app for fishermen*. Retrieved June 28, 2021, from https://www.machli.reliancefoundation.org/

Shellcatch. (2019). *e-reporting: From paper to digital*. https://www.shellcatch.com/welcome/ereporting/

Singh, D. (2018). *mKrishi Fisheries: Sea in Your Hand*. Impakter. https://impakter.com/mkrishi-fisheries-sea-hand/

SINTEF. (2016). *FiskInfo app*. https://www.sintef.no/siste-nytt/2016/fiskinfo-app/

Siriraksophon, S., Rukjai, P., Konphet, P., & Imsamrarn, N. (2017). *Automating Marine Fisheries Catch Documentation Schemes: the eACDS*. http://163.44.197.130/eACDS/.

*Straight off the boat*. (2021). https://straightofftheboat.com/

Takemura, S., Kogushi, S., Wells, M., & Makino, M. (2019). The PICES-MAFF sponsored project on “Building Capacity for coastal monitoring by local small-scale fishers” (FishGIS): Mobile phone-based monitoring technology and training workshop. *PICES Press: Newsletter of the North Pacific Marine Science Organisation*, *27*(1), 16–18.

TrazApp. (n.d.). *TrazApp - Digital solutions for fishing*. Retrieved September 23, 2021, from https://www.trazapp.org/

Vericatch. (2020a). *Electronic Logbooks (ELOGs) For Canadaian Fisheries*. https://vericatch.com/products/electronic-logbooks/

Vericatch. (2020b). *FisheriesApp - The Leading Solution in Fisheries Catch Reporting*. https://vericatch.com/products/fisheriesapp/

Vericatch. (2020c). *KnowYour.Fish: Seafood Traceability Software*. https://vericatch.com/products/knowyourfish/

WCPFC. (2020). *Guide to the WCPFC High Seas Transhipment Electronic Reporting System*. https://www.wcpfc.int/doc/tser-user-guide/guide-wcpfc-high-seas-transhipment-electronic-reporting-system

Wiyono, E. S., Raharjo, S. S. S., & Permana, S. M. (2018). *Fishermen acceptance on introduction of fishing technology: perception and its development strategies* (Vol. 11). http://www.bioflux.com.ro/aacl

WWF. (2014). *Happy Fish, Happy People*. https://www.worldwildlife.org/stories/happy-fish-happy-people

Xesmar. (2019). *Plataforma tecnolóxicapara a xestión dos traballos do mar*. https://www.xesmar.es/#!/
